# Skating on thin ice: pragmatic prescribing for medication refractory schizophrenia

**DOI:** 10.1186/s12888-015-0559-x

**Published:** 2015-07-25

**Authors:** Derek K. Tracy, Dan W. Joyce, S. Neil Sarkar, Maria-Jesus Mateos Fernandez, Sukhwinder S. Shergill

**Affiliations:** 1 Oxleas NHS Foundation Trust, Green Parks House, Orpington, Kent, BR6 8NY, London, UK; 2 Cognition, Schizophrenia & Imaging Laboratory, Department of Psychosis Studies, the Institute of Psychiatry, King’s College London, London, UK; 3 South London and Maudsley NHS Foundation Trust, London, UK; 4 Central and North West London NHS Foundation Trust, London, UK

**Keywords:** Schizophrenia, Psychosis, Refractory, Treatment

## Abstract

**Background:**

Clozapine is the treatment of choice for medication refractory psychosis, but it does not benefit half of those put on it. There are numerous studies of potential post-clozapine strategies, but little data to guide the order of such treatment in this common clinical challenge. We describe a naturalistic observational study in 153 patients treated by a specialist psychosis service to identify optimal pharmacotherapy practice, based on outcomes.

**Methods:**

Medication and clinical data, based on the OPCRIT tool, were examined on admission and discharge from the national psychosis service. The primary outcome measure was the percentage change in mental state examination symptoms between admission and discharge and the association with medication on discharge. Exploratory analyses evaluated the specificity of individual medication effects on symptom clusters.

**Results:**

There were fewer drugs prescribed at discharge relative to admission, suggesting an optimisation of medication, and a doubling of the number of patients treated with clozapine. Treatment with clozapine on discharge was associated with maximal decrease in symptoms from admission. In the group of patients that did not respond to clozapine monotherapy, the most effective drug combinations were clozapine augmentation with 1) sodium valproate, 2) lithium, 3) amisulpride, and 4) quetiapine. There was no support for a dose–response relationship for any drug combination.

**Conclusions:**

Clozapine monotherapy is clearly the optimal medication in medication refractory schizophrenia and it is possible to maximise its use. In patients unresponsive to clozapine monotherapy, augmentation with sodium valproate, lithium, amisulpride and quetiapine, in that order, is a reasonable treatment algorithm. Reducing the number of ineffective drugs is possible without a detrimental effect on symptoms. Exploratory data indicated that clozapine was beneficial across a range of symptoms domains, whereas olanzapine was beneficial specifically for hallucinations and lamotrigine for comorbid affective symptoms.

## Background

Clinical outcomes in schizophrenia are often disappointing: a third of patients are resistant to standard pharmacological interventions [1], fewer than one in eight individuals fully recover [2], and data are particularly disheartening for negative and cognitive symptoms [3]. Clozapine is the most effective antipsychotic for more treatment naïve [4] and medication refractory patients [5], but half will not show significant improvement on it [6–9]. There is limited evidence for polypharmacy – including augmentation of clozapine [10] - or above licensed dose use of antipsychotics, and both increase side effects [11]. Naturalistic – or partially naturalistic - studies such as CATIE and CUTLASS confirmed clozapine’s superior efficacy (although this was not the primary outcome for the CATIE study, and assignment to clozapine in the phase II part was not randomised), and the absence of any significant effect on negative or cognitive symptoms; but were able to offer little pragmatic guidance for post-clozapine treatment. Guidelines [12–14] provide extensive lists of potential post-clozapine strategies, including the use of other drug classes including mood-stabilisers and anti-depressants - but recognise the frank inability to select between them. Clinicians are thus faced with an unenviable but relatively common challenge of treating patients resistant to first and second line treatments in the absence of robust data to further guide them [15].

Three critical issues occur in the “post-clozapine” literature: study methodologies; heterogeneity of psychosis outcomes; and a medication accumulation bias. The research gold standard remains the double-blinded randomised controlled trial (RCT), but trial participants are less representative of clinical populations with respect of co-morbidity, insight and capacity to consent, and anticipated drug class effects are underpowered to elicit the well-recognised subpopulations of full-, partial-, and non-responders. Trials typically evaluate a single compound against placebo, with fewer head-to-head trials, and very few data on adjunct treatments. It is perhaps not surprising that there is little agreement about clinical, pharmacological or demographic factors associated with outcomes [16]. Clinical opinion with respect to individual patient presentations – that is specific symptom profiles and local prescribing culture - often dictates choice and duration of treatment, with wide geographical variation [17], often contrary to evidence [18]. Finally patients typically accrue increasing medications, with management often comprising addition of new medication, with existing (or previously added) ones seldom discontinued for fear of causing destabilisation. However remission often does not occur and such polypharmacy is more likely to become the rule rather than the exception.

### Aim

We describe a naturalistic study of outcomes in 153 treatment refractory inpatients on a specialist tertiary psychosis service, the National Psychosis Service (NPS) at the Maudsley and Bethlem Royal Hospitals, London, UK. The admission criteria to the service are analogous to those of the United Kingdom’s Department of Health definition and guidelines [19] for specialist services (Table 1). Such criteria give broad descriptions of how a treatment refractory illness might manifest, for example through high symptom burden and/or significant impact on social functioning, as well as illustrate anticipated prior pharmacological and psychological interventions and durations. However they act as guidance, and can be superseded by the clinical judgement of the referring or assessing psychiatrist; for example, whilst it would ordinarily be anticipated that a referred patient would have had previous psychological intervention(s), it is recognised that there can be circumstances wherein this would have been untenable; furthermore “high symptom burden” and “significant impact on functioning” are not further quantified, but designed to allow a clinical discretion in interpretation. We have previously reported upon the nature of, and the clinical and psychosocial improvements demonstrated by, this service [20], but briefly, the National Psychosis Unit is a 23 bedded unit, staffed with Consultant Psychiatrists, junior doctors, specialist clinical psychologists, mental health nurses, a social worker and occupational therapy staff members. Staff work closely within an interdisciplinary setting with weekly in-depth multiprofessional ward reviews of each patient. All patients are offered specialist psychological input, family therapy, and a range of occupational therapy. It is sited within the Bethlem Royal Hospital, South London, in extensive landscaped grounds, with access to swimming, a gym, and a range of occupational therapy activities. There is ready access to specialised pharmacy, haematology, and cardiology input, and it has a close association with the Psychosis Clinical Academic Group at the Institute of Psychiatry, Psychology and Neuroscience, King’s College London, enabling very close links with field-leading research. Herein we set out to evaluate: a) the feasibility of rationalising pharmacological treatment; b) to identify the most common medications prescribed on discharge (as a posited index of their optimal tolerated treatment); c) exploratory analysis of medication(s) associated with overall outcomes and specific symptom domains.

**Table 1:** Criteria for referral to the NPS, based on Department of Health Guidelines (DoH, 2009)

Criteria for complex and/or refractory disorder services	
Generic complex/refractory criteria	Specific to a psychosis centre
Diagnostic uncertainty hampering treatment	Failure to respond adequately (or tolerate) two antipsychotics (at least one atypical)
Persistently high symptom burden	
Significant impact on functioning	Attempted adequate trial of clozapine, usually for a minimum of 6–9 months
Persisting (>2 years) pattern of incapacity despite appropriate treatment	
Multiple comorbidities increasing likelihood of chronicity	Appropriate psychological therapies such as cognitive behavioural therapy and family interventions should have been attempted
Need for specialised treatments, e.g. TMS	
Inpatient stay >6-12 months	

## Methods

The methodology of data collection is described in our earlier work [20], but in brief, clinical notes of patients admitted to the NPS between 2001 and 2007 were collated and retrospective analysed using the OPCRIT system. Ethical approval for such retrospective analysis of patient case notes was approved by the Research and Development office of South London and Maudsley NHS Foundation Trust, UK. Of the 182 patients admitted during this time, 153 case notes (86 male, 67 female) were deemed to contain sufficient information to allow reliable assessment of clinical information: all patients with such information were included in the study, and no other inclusion or exclusion criteria were utilised. Mean participant age at the point of admission was 33 (s.d. = 10.9); mean duration of admission was 254 days (s.d. = 169). All participants met ICD-10 criteria for a primary diagnosis of schizophrenia, which was by definition of admission to this tertiary unit, considered considerably treatment refractory; 36 had a comorbid personality disorder; 36 had a lifetime history (and 24 at the point of admission) of alcohol dependency or harmful use; 49 had a lifetime history (and 37 at the point of admission) of cannabis dependency or harmful use; and 31 a lifetime history (23 at the point of admission) of dependency or harmful use of other substances. These categories are not exclusive, with some individuals having more than one co-morbidity. The only exclusion criterion for patient admission to the National Psychosis Unit is if they cannot be physically managed by staff due to risk of significant agitation or violence.

The OPCRIT is a reliable and validated tool [21] utilising an inventory of psychopathological symptoms, demographics, and disease course variables that are scored, with algorithms for clinical diagnosis [22]. The notes on admission to, and discharge from, the NPS were assessed to yield OPCRIT mental state examination (MSE) severity scores for each time point across 5 domains: affective symptoms, abnormal perceptions, abnormal beliefs, speech and thought disorders, and appearance and behaviour. Demographic information was collated for all participants; and medications, including dose, on admission and discharge, were recorded.

The primary measures of outcome were change in mental state examination (MSE) symptom severity measured as the sum of the 5 OPCRIT mental state variables. Each symptom domain score is ordinal (with zero indicating an absence of those symptoms and higher values indicating increasing symptom severity) with maximum values as follows: affective symptoms (maximum score = 58), abnormal perception (12), abnormal belief (41), speech and thought disorder (13), and appearance and behaviour (24); resulting in a maximum summed symptom severity of 148. The primary outcome was the percentage change in total 5-domain MSE symptom score at admission and discharge (for example, if the total score at admission is 95, and at discharge 55, this represents a percentage improvement of 42 %). We also performed analyses on this primary outcome for clozapine monotherapy, and clozapine augmented with the two most frequently prescribed (in our sample) antipsychotics (quetiapine, amisulpride) and mood stabilisers (lithium, valproate). For these analyses, a simple linear regression was used to study the effect of each drug, dose and interactions between drugs/dose on the primary outcome. Pre-processing and data quality checking were performed using MATLAB 2013a. Tabulated data for each analysis was then exported for graphing using the R statistical package with ggplot2 libraries.

For the exploratory analysis of potential drug effects on specific symptom domains we evaluated if the six most commonly prescribed drugs - on which we had enough data to model – produced changes in OPCRIT outcomes, which we defined categorically as: 1, worse that average improvement from admission to discharge, where average was defined as the median change for all patients during this time; 2, average improvement during this time; 3, better than average improvement. Patients who scored worse than the median - IQR (interquartile range) get assigned outcome 1 for that mental state outcome (e.g. speech and thought); those who scored median +/− IQR got assigned 2; and those who outperformed and scored over the median + IQR got outcome 3. In this subanalysis each patient therefore had an ordinal, categorical score (valued at either 1, 2 or 3) in the five outcome domains (the OPCRIT domains relating to mental state). Conventional multiple regression analysis can only model univariate or continuous outcomes, whereas we aimed to explore the relationship between *multiple* outcomes and multiple drug/drug combinations. To proceed, we constructed five separate ordinal probit regression models, one for each of the mental state categorical outcomes with the same drug combinations as predictors. This effectively represents five sub-analyses on the *same* predictors but with *different* outcomes, there is a risk of Type II error, where a significant association between drug combinations (i.e. regression coefficients with *p* values less than 0.05) is found purely by chance because of the number of sub-analyses conducted. To counter this, the fitted regression coefficients for each of the five ordinal probit regressions were tabulated together in descending rank order of their p-values. These augmented regression coefficients were then subjected to false-discovery rate (FDR) corrections using the Benjamini and Hochberg method to control the Type II error rate. We then report only associations where regression coefficients survived FDR correction at the q < 0.2 level appropriate for exploratory analyses.

From the small subset of significant results, we then transformed the regression coefficients to probabilities of being in the worse, average or better than average categories for each drug combination in each of the five mental state domains. The advantage of this method is that it captures associations between drug combinations and changes over the mental state domains similarly to multivariate pattern classification methods, while retaining the ability to draw inferences about effect sizes in an interpretable fashion (e.g. by extracting regression coefficients that quantify the strength and direction of the association with mental state domains).

### Missing data

Of 153 records, 11 patients had one missing drug dose value for medications on discharge and one patient was missing 2 dose values. In total, 3.5 % (145 individual values from a total of 4131) of the demographics data had missing values. There was no systematic relationship between these missing values; they were treated as missing-at-random and non-parametric imputation by *k*-nearest neighbours was used to impute missing values.

## Results

1. The prescribing data at discharge.

Table 2 gives details of medication at admission and discharge; 47 patients were treated with clozapine (median dose 450 mg) on admission, increasing to 90 on discharge (412.5 mg). Of the 47 admitted on clozapine, 41 remained on clozapine at discharge; thus 49 patients were newly commenced on this drug during admission. The next most frequently prescribed medications at discharge were sodium valproate (37/153, 1325 mg/day), amisulpride (33/153, 400 mg/day), olanzapine (25/153, 20 mg/day), lithium (21/153, 800 mg/day), quetiapine (21, 600 mg/day) and lamotrigine (18, 200 mg/day). Other medications prescribed in fewer than 8 individuals were: risperidone consta, zuclopenthixol, risperidone, carbamazepine, sulpiride, melperone, haloperidol, droperidol, flupenthixol, chlorpromazine, valproic acid, pipotiazine, and perphenazine. These figures do not account for polypharmacy in individual patients, and only reflect total medications used.

**Table 2:** Medications on admission to, and discharge from, the National Psychosis Service

Drug	Admission			Discharge		
	Frequency	Median dose	IQR	Frequency	Median dose	IQR
Clozapine	47	450	218.8	90	412.5	275
Sodium valproate	28	1200	925	37	1325	1000
Amisulpride	20	600	600	33	400	325
Olanzapine	36	20	2.5	25	20	10
Lithium	11	800	0	21	800	200
Quetiapine	15	550	325	21	600	237.5
Lamotrigine	10	200	25	18	200	50
Risperidone consta	8	2.7	0.5	7	2.7	0.9
Zuclopenthixol	14	28.6	14.3	6	17.9	28.6
Risperidone	11	4	2	5	4	2
Carbamazepine	11	400	350	4	400	200
Droperidol	2	90	120	3	40	0
Sulpiride	7	800	800	3	800	1200
Haloperidol	8	10	0	3	10	3.8
Melperone	1	250	0	3	500	75
Flupenthixol	6	4.4	2.9	2	4.5	3
Chlorpromazine	6	175	200	2	800	0
Valproic acid	4	750	250	1	1500	0
Perphenazine	0	0	0	1	24	0
Pipotiazine	5	6	4.7	1	5.3	0
Aripiprazole	3	30	0	0	0	0
Fluphenazine	2	1.8	0	0	0	0
Trifluoperazine	1	20	0	0	0	0

### Monotherapy prescribing at discharge: clozapine and olanzapine

**Fig. 1:**
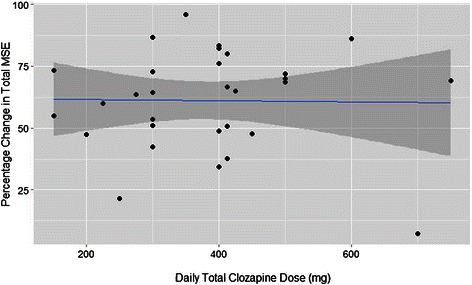
Outcome with Clozapine Monotherapy, with black filled circles each representing a single patient. The blue line is the linear regression trend line, with the dark grey area illustrating the 95 % confidence interval

The most common monotherapeutic antipsychotics at discharge were clozapine (33/153) and olanzapine (8/153). With clozapine (median monotherapy dose 400 mg/day (IQR = 121.9 mg)) there was a median improvement in symptoms (percentage change in total MSE variables) of 64.6 % (IQR = 23.8 %) (see Fig. 1). For olanzapine monotherapy (median dose 15 mg, IQR = 5 mg) median improvement was smaller at 30.5 %, with wider variation (IQR = 56.2 %). In both drugs there was no significant relationship between the prescribed dose and improvement.

### Clozapine co-prescribing at discharge

**Fig. 2:**
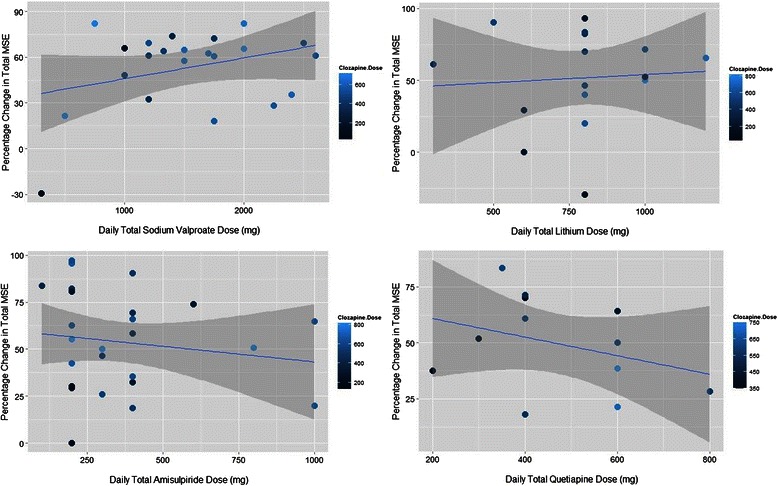
Clozapine co-prescribing with the most efficacious agents. Filled circles each represent a single patient, with the dark to light blue gradient representing lower to higher clozapine doses. The blue line is the linear regression trend line, whilst the dark grey area represents the 95 % confidence interval. Data are shown for co-prescribing with sodium valproate (top left), lithium (top right), amisulpride (bottom left), and quetiapine (bottom right)

The most common coprescribed antipsychotics were amisulpride and quetiapine respectively; and mood stabilising agents were sodium valproate, lithium and lamotrigine. Table 3 summarises the outcomes and median dose used for the different combination therapies, and Fig. 2 illustrates the efficacy data of the four most efficacious combinations. For clozapine + amisulpride, the median improvement was 53.0 % (IQR = 39.5 %). For clozapine + quetiapine, a similar pattern was found with median improvement of 51.0 % (IQR = 30.5 %). No dose–response relationship or interaction was determined for any combination. With sodium valproate (median improvement 62.5 % (IQR = 21.1 %)) an apparent trend in the data is driven by an outlier (visible on the bottom left), and when this is removed, there is no significant dose–response effect or interaction between drug doses and response. Of note, a majority of patients (15) responded with total daily valproate doses in the range 1000-2000 mg. For the 16 individuals on combinations of clozapine + lithium, the median improvement was 56.9 % (IQR = 36.8 %) and again, there was no significant dose–response or interaction effect (with the caveat that serum lithium levels were not available and only total daily dose was measured) but median clozapine doses were lower than other augmentation strategies. Of the 14 patients on clozapine + lamotrigine, a median improvement of 43.7 % (IQR = 32.05) was demonstrated. No dose–response or interaction effects were statistically significant.

**Table 3:** Clozapine co-prescribing with antipsychotics and mood stabilisers (ranked in descending order of percentage median improvement change on MSE symptom severity scores)

Augmentation	Patients	Median clozapine dose (mg)	Clozapine interquartile range (mg)	Median co-prescribed dose (mg)	Co-prescribed dose interquartile range (mg)	Median improvement %	Improvement IQR %
Sodium valproate	22	550.0	225.0	1500	800.0	62.5	21.1
Lithium	16	375.0	375.0	800	100.0	56.9	36.8
Amisulpride	26	500.0	237.5	300	200.0	53.0	39.5
Quetiapine	13	550.0	159.4	400	212.5	51.0	30.5
Lamotrigine	14	337.5	262.5	200	37.5	43.7	32.0

### Non-clozapine combination therapy at discharge

Two ‘non-clozapine’ combinations, olanzapine + valproate (8 patients) and quetiapine + valproate (6 patients), occurred sufficiently commonly to allow outcome analyses. With the former, the median improvement was substantially lower at 15.4 % (IQR = 31.8 %), with: a trend toward lower doses of olanzapine and better outcomes (but no statistically significant dose–response interactions); no evidence that doses of olanzapine greater than 20 mg are beneficial (median = 22.5 mg, IQR = 22.5 mg); but wide variation in valproate doses (median = 1600 mg, IQR =1150 mg). Six individuals were on the quetiapine + valproate combination, and there was a median improvement of 33.0 % (IQR = 27.3 %) and again, no significant dose–response/interaction effects. The median quetiapine dose was 700 mg (IQR = 350 mg) with a median sodium valproate dose of 1675 mg (IQR 825 mg). Table 4 charts final describing choices against patient demographic factors.

**Table 4:** Final prescribing patterns charted against patient variables, including age, sex, a positive family history of a psychotic illness (FHx), a concomitant personality disorder (PD), alcohol, cannabis, or other illicit drug use

Rx: clozapine	Age median (IQR)	Sex (M/F)	FHx	PD	Alcohol	Cannabis	Other substances
Monotherapy	27 (16)	23/13	5	13	11	13	10
+ Quetiapine	37 (12)	8/5	2	7	2	4	4
+ Amisulpiride	31 (7)	16/10	4	5	5	7	8
+ Lithium	30 (7)	8/8	1	4	2	3	2
+ Valproate	33 (7)	10/12	5	5	7	11	9

2. Exploratory analyses of medication effects on specific symptom subdomains

Table 5 shows the results of the multivariate analyses of medication combinations and change in mental state domain categories. From this, the general trend of polypharmacy having worse outcomes is replicated, and clozapine monotherapy is again associated with (at worst) being a member of the average improvement category on each mental state variable, and at best abnormal belief, perception and speech and thought domains show improvement in the above average category. Clozapine + quetiapine as dual therapy appears associated with worse than average outcomes (which likely reflects the principle of poorer outcomes in polypharmacy than a deleterious combination of medications). A similar trend is shown for clozapine + lamotrigine. Finally, quetiapine + valproate combined confer group-average improvement on appearance and behaviour.

**Table 5:** Exploratory multivariate analyses of medication on change in mental state domains

	Appearance and behaviour	Abnormal belief	Abnormal perception	Affect	Speech and thought
Clozapine monotherapy	*0.0012*	**0.0004**	**0.0080**	*0.0260*	**0.0031**
Clozapine & quetiapine	0.0086	0.0267	-	-	-
Clozapine & lamotrigine	-	-	-	-	0.0136
Quetiapine & valproate	*0.0389*	-	-	-	-

Values indicate the p-values of the drug combination as predictor for change in categorical mental state variable at discharge (only those surviving FDR correction at the q = 0.20 level are shown). Bold values indicate medication (combination) associations with mental state domain where improvement above the group average was found (i.e. patients on this combination would be more likely to be in the above average category for improvement). Italicised values show the same information but where the highest probability was for the “average” change category. Underlined values show where the medication (combination) was associated with a worse than average change in that mental state domain

The treatment protocol directs treatment to disabling symptoms, agnostic to categorical (ICD, DSM) disease category and therefore, we did not code schizoaffective patients separately from, for example, paranoid schizophrenia. The characteristics of schizoaffective patients compared with schizophrenia in terms of demographics and comorbidity has recently been documented by Pagel et al. [23]. In their meta-analysis, they compared 2684 people with a schizoaffective diagnoses, against 10814 patients diagnosed as having schizophrenia and showed that the schizoaffective group contained a higher proportion of females (52 %), a younger mean onset of illness of 23.3 (standard deviation +/− 3.8 years) with higher ratings of both psychotic and affective symptoms. It is therefore important and informative to examine the proportions of patients with a significant affective component (as compared to disorganised thought and speech, abnormal belief and perception) in our treatment refractory sample. Given all patients in our sample had high psychotic symptom loads, we further divided our data on the basis of affective symptoms as follows. The distribution of admission OPCRIT mental state variables significantly deviated from normality, so we computed the non-parametric Tukey five-number summary [24] yielding the median and upper-hinge which, in our sample, correspond to the 50th (median) and 75th percentile of the distribution of affective symptom load of the OPCRIT. We then analysed the demographics of the subsample with the highest affective symptom load, i.e. those with affective symptom load greater than 75th percentile/upper-hinge and compare with those below the 75th percentile. We found that 37/153 patients had affective symptom load greater than the 75th percentile. The median age of onset was 32 (IQR = 16) and 31.5 (IQR = 15.25) in the high and low affective symptom group respectively. In terms of gender proportions, in the high symptom load, 46 % were male and 54 % female, consistent with Pagel et al. [23]. In the lower affective symptom group, the proportions were 57 % male and 44 % female. We then summed symptom load on psychotic symptoms of speech and thought disorder, abnormal perception and belief for the high affective symptom group and compared them to the lower affective symptom group. A Kolmogorov-Smirnov test confirmed that the total psychotic symptom load differs between the high affective compared to the low affective symptom group (D = 0.419, p < 0.001) with the high affective symptom group having higher median summed psychotic symptom load of 29 (IQR = 15) compared to the low affective group with median 19.5 (IQR = 16.3). In summary, when compared with the findings of Pagel et al. [23], we found 37 / 153 patients had similar profiles to schizoaffective patients (versus schizophrenia without affective symptoms) with the exception that in our sample, the median age of onset was similar between the two groups.

## Discussion

The optimal management of treatment refractory schizophrenia poses a significant personal, social and economic challenge, and there is a dearth of evidence to support treatment beyond clozapine monotherapy. We herein report the results of a naturalistic study of 153 patients on a tertiary psychosis unit. No specific treatment algorithm was utilised, as the aim was to describe ‘real-world’ prescribing patterns that altered in accordance to patient response rather than study design, which might capture a wider and more representative cohort of refractory patients (including many with significant histories of substance misuse and Axis II disorders); and that we might utilise clinical outcomes as one additional element to guide prescribing. Patients are typically discharged from the NPS when they are at their most stable, and we accepted a model that that discharge medication represented their optimal treatment to inform further future treatment protocols.

Rational prescribing in medication refractory individuals would maximise the use of clozapine, and remove any medication without clear benefit. Clozapine use almost doubled from admission, with just over a third on monotherapy; and in those few patients intolerant or unwilling to comply with clozapine, the only other monotherapeutic treatment of note was olanzapine (n = 8). At discharge, there were 9 different drugs being used by 5 or more patients (excluding clozapine), while the analogous figure at admission was 16, suggesting a rationalisation and reduction in the range of non-effective drugs accrued in these patients. Interestingly, there was a decrease in the use of depot antipsychotic medication from 29 to 13 at discharge that may reflect the patients transferred to clozapine. 38/153 patients with medication refractory illness were discharged from the NPS on monotherapy, compared with 18 on monotherapy on admission, and the pattern was still of significant clinical improvement. Rationalisation of medication load, without clinical destabilisation of patients’ mental states, thus appeared to be an achievable aim.

Unsurprisingly co-prescribing with clozapine was far more common than other non-clozapine combinations. If our assumption is correct that the discharge medication represents individuals’ optimum prescribing, these combinations would be a proxy marker for efficacy and were likely to be associated with positive clinical benefit. We examined the extent of improvement in mental state parameters as a function of discharge medication. Clozapine effected the greatest improvements in OPCRIT markers, with a median symptom improvement of 64.6 % (IQR = 23.8 %). After clozapine monotherapy the greatest median improvement was seen with the addition of the mood stabilisers sodium valproate (median improvement 62.5 %) and lithium (56.9 %) respectively, with the former having the narrowest improvement IQR of any added drug. Following this the next most efficacious additions were the antipsychotics amisulpride (53 %) and quetiapine (51 %), though the absolute difference in median improvements from the mood stabilisers was not large. There were far fewer ‘non-clozapine’ combinations, and the only ones with sufficient numbers to meaningfully analyse contained the mood stabiliser sodium valproate and an antipsychotic (olanzapine and quetiapine), with very limited improvements in mental state relative to the clozapine combinations.

Overall there was no evidence for dose–response in any drug or combination of drugs. There were increased median plasma clozapine levels in the combination therapy relative to clozapine monotherapy. However, this is likely to be an artefact of the treatment regimes used on the unit, where the policy is to: aim for plasma levels of 0.35 mg/l in the first instance; to increase the dose to levels of 0.50 mg/l if there is an inadequate response; and if there is still insufficient benefit to augment with another medication.

The exploratory analysis of the individual drug effects on specific symptom domains demonstrated the superiority of clozapine in three domains (speech and thought; abnormal beliefs; and appearance and behaviour), and, with olanzapine, showing the largest effect in “abnormal perception”; supporting the research data on clozapine treatment of medication refractory psychosis. There was a notable potential beneficial effect of treatment with lamotrigine on patients with an affective symptoms cluster, although this did not match the overall effectiveness of clozapine combination treatment with other mood stabilisers.

Caveats with our naturalistic study include that this was an exploratory analysis in a selected sample, in the absence of a control group and any blinding of assessments. As such it cannot be regarded as evidence based as would occur within the context of a Randomised Controlled Trial. A limitation is that it leaves unanswered the question of whether the apparent advantages of some treatment strategies were dictated by the intervention, or if it was the illness state the governed the choice of intervention. Table 4 charts individuals’ demographic data against prescribing choice, but participant numbers mean that firm conclusions cannot be reached on this important issue. However to go beyond descriptive to inferential analyses would have required either combinatorially large, multisubgroup analysis using linear/generalised models, or the development of a complete model of proposed statistical relationships (with hypothesised dependencies between these variables) for the 90+ OPCRIT variables (predictors and outcomes). The latter, more elegant and complete, option represents our group’s ongoing research, but the methodology has yet to fully mature, and is beyond the scope of this paper on pragmatic prescribing.

The OPCRIT is a reliable tool, but factorial validity does not confer construct validity, and factor analyses of the OPCRIT do not necessarily support neo-Kraepelinian assumptions about diagnostic categories. However, the value of OPCRIT is its mobility between categorical and nomothetic dimensions, and OPCRIT has been validated insofar as when the multidimensional data are parsed by algorithms for categorical diagnosis, there is concordance between clinician-assigned diagnostic categories, ICD-10 and DSM. An advantage to use of OPCRIT is that the mental state domains offer a symptom load score which is agnostic to specific diagnostic category or sub-category (e.g. paranoid versus hebephrenic schizophrenia) and treatment refractoriness. Future work might appropriately attempt to replicate these findings utilising other outcome measures, not least with more functionally meaningful tools assessed in real-world settings beyond the inpatient ward. Such work would better align with individuals’ typical goals within a recovery model beyond symptom reduction.

Retrospective note analysis is also open to challenge [25] including through inadvertent biases in those collecting the data. Our data was rated by two psychiatrists trained in the use of OPCRIT, and a test-retest on a random sample of ten note sets showed good inter-rater reliability [20]. Further, in the unit assessed, as is the case in most UK inpatient wards, much of the documentation is carried out by junior doctors who change every six months: in view of this and the inconsistent use of clinical scales over the time period it was considered that the only valid and reliable way to obtain clinical information was thus through the use of an operationalised system such as OPCRIT. Adherence is a critical factor to consider in medication refractory patients, and the literature on this topic is disheartening [26]. Many will suboptimally adhere, which may adversely affect their recovery, though there is a very large range of behaviour and outcomes covered by the construct of 'adherence'. Adherence to treatment does not form part of the explicit referral criteria or guidelines, and it is expected that referring clinicians will have considered and, where relevant, tried to manage this issue. Inpatient units will have better opportunities to monitor adherence, and this fact might have affected our outcomes; adherence was not measured before or after treatment, and rates of adherence during inpatient admission are not reported. However, referrers would have had access to local, non-specialised, inpatient units for admission and monitoring of adherence had this been considered a critical factor. There is considerable variation in the use of long acting injectable (LAI or ‘depot’) medication geographically and between individual services; it could be argued that fewer than expected individuals with medication refractory illnesses were on this treatment modality at the time of referral.

These data do not take into account the psychological, occupational therapeutic, and nursing care provided for patients on this unit, and furthermore the provision and nature of these services on a tertiary unit might not be reflective of wider practice and staff availability. The National Psychosis Unit is undoubtedly an enriched environment with highly trained and motivated staff with specialist skills in psychosis management, and ready access to rapid interdisciplinary care and treatment. Finally, the nature of our data collection meant that we could not ascertain if any medication changes were made to reduce or avoid drug side-effects or risks, which is a potential biasing factor when interpreting these data.

## Conclusions

In summary, our data show that medication can be optimised in a chronic medication refractory sample of patients with schizophrenia; and that this is associated with significant clinical improvement in specific symptom domains. Reduction in the range of medication used did not result in a destabilisation in mental state, but was associated with positive outcomes. The general principles to be drawn from the results suggest primary use of clozapine therapy where possible in medication refractory schizophrenia. Research [27] has demonstrated a mean delay of over four years before clozapine is commenced, and whilst there are many reasons why this might occur, our results reinforce the need to actively target and overcome obstacles to clozapine treatment in medication refractory populations. These would include rechallenging a patient after a “red result”, liaising with colleagues in cardiology and haematology to monitor and treat in the many cases with comorbid illness, either iatrogenic or extant conditions; and aggressively treating any side effects such as sedation, hyper-salivation, and tachycardia. Careful identification and management of comorbid symptoms such as OCD and mood disorders is also necessary – with regular monitoring of these target symptoms – allied to a clear plan for discontinuing any medication that does not benefit the target symptoms.

Given the caveats of all observational studies, our data suggests that if clozapine monotherapy is ineffective, that augmentation with sodium valproate is the most efficacious option, followed by augmentation with amisulpride, lithium, quetiapine, and then lamotrigine. The last of these, lamotrigine, whilst proving the least efficacious in combination with clozapine, is noteworthy for demonstrating superior efficacy in managing affective symptoms.

## Abbreviations

MSE: Mental state examination
OCD: Obsessive compulsive disorder
