# Correction to: Skating on thin ice: pragmatic prescribing for medication refractory schizophrenia

**DOI:** 10.1186/s12888-019-2155-y

**Published:** 2019-05-27

**Authors:** Derek K. Tracy, Dan W. Joyce, S. Neil Sarkar, Maria-Jesus Mateos Fernandez, Sukhwinder S. Shergill

**Affiliations:** 1 Oxleas NHS Foundation Trust, Green Parks House, Orpington, London, Kent BR6 8NY UK; 2 Cognition, Schizophrenia & Imaging Laboratory, Department of Psychosis Studies, The Institute of Psychiatry, King’s College London, London, UK; 3 South London and Maudsley NHS Foundation Trust, London, UK; 4 Central and North West London NHS Foundation Trust, London, UK

**Correction to: BMC Psychiatry (2015) 20:48**

**https://doi.org/10.1186/s12888-015-0559-x**

After publication of the original article [1], the authors have notified us that there was an oversight on acknowledging funding received for the study. They would like to mention that Professor Sukhi Shergill was funded by an ERC Consolidator Award.
